# Values Underpinning Integrated, People-Centred Health Services: Similarities and Differences among Actor Groups Across Europe

**DOI:** 10.5334/ijic.6015

**Published:** 2022-08-08

**Authors:** Nick Zonneveld, Ludo Glimmerveen, Patrick Kenis, Nuria Toro Polanco, Anne S. Johansen, Mirella M.N. Minkman

**Affiliations:** 1 Tilburg University, Department of Organization Studies, Warandelaan 2, 5037 AB Tilburg, The Netherlands; 2 Vilans, National Centre of Expertise in Long Term Care, Utrecht, PO Box 8228, Churchilllaan 11, 3503 RE Utrecht, The Netherlands; 3 Vrije Universiteit, Department of Organization Sciences, Amsterdam, The Netherlands; 4 Tilburg University, Tilburg School of Economics and Management (TiSEM), Tilburg, The Netherlands; 5 WHO, Headquarters, Geneva, Switzerland; 6 WHO Regional Office for Europe, Copenhagen, Denmark; 7 Vilans, National Centre of Expertise in Long Term Care, Utrecht, The Netherlands; 8 TIAS School for Business and Society (TiU), Tilburg, The Netherlands

**Keywords:** integration, integrated care, health services, Europe, policy, organisation

## Abstract

**Introduction:**

In addition to the functional aspects of healthcare integration, an understanding of its normative aspects is needed. This study explores the importance of values underpinning integrated, people-centred health services, and examines similarities and differences among the values prioritised by actors across Europe.

**Methods:**

Explorative cross-sectional design with quantitative analysis. A questionnaire of 18 values was conducted across Europe. A total of 1,013 respondents indicated the importance of each of the values on a nine-point scale and selected three most important values. Respondents were clustered in four actor groups, and countries in four European sub-regions.

**Results:**

The importance scores of values ranged from 7.62 to 8.55 on a nine-point scale. Statistically significant differences among actor groups were found for ten values. Statistically significant differences across European sub-regions were found for six values. Our analysis revealed two clusters of values: ‘people related’ and ‘governance and organisation’.

**Discussion and conclusion:**

The study found that all 18 values in the set are considered important by the respondents. Additionally, it revealed distinctions in emphasis among the values prioritised by actor groups and across sub-regions. The study uncovered two clusters of values that contribute to a conceptually based definition of integrated, people-centred health services.

## Introduction

An increasing number of individuals with multiple, complex conditions require services from numerous professionals, spanning disciplines and sectors. A ‘solution’ for this situation is often found by connecting and co-ordinating healthcare services, a process frequently referred to as integrated care [1,2]. The integration of healthcare services is widely seen as a promising strategy in the provision of good quality person centred care [3,4,5]. In practice, however, healthcare services are still fragmented in many countries [6,7,8]. The recent COVID-19 crisis has accentuated this fragmentation in healthcare services across the globe [9]. Responses are often not integrated, leading to poor health outcomes and greater inequality across populations [10].

The existing literature on integrated, people-centred health services often describes technical interventions, such as case-management [11], payment models [12,13] and information technology [14]. In the Rainbow Model of Integrated Care (RMIC), Valentijn refers to these aspects as key components of functional integration [15]. However, if health services integration only involves functional aspects, then why is it so difficult to implement [14,16,17,18] and so hard to demonstrate improved outcomes [19,20,21,22,23,24,25]?

The risk of mainly focusing on technical factors is that possible ‘softer’ human aspects may be glossed over. In the RMIC, these aspects are referred to as normative integration [15]. This is relevant because, in addition to technical activities, the delivery of integrated, people-centred health services consists of several interactive processes that often take place in multi-level networks, among a variety of actors with different backgrounds, who are influenced by numerous contextual factors [15,26]. Goodwin [27] describes this situation as a complex black box.

A better understanding of the factors that contribute to these complexities could provide important information in support of the integrated, people-centred health services agenda. In recent years, there has been growing evidence about the importance and the role of normative dimensions of integrated, people-centred health services, such as communication [28], relationship dynamics [29], trust [30], emotional dimensions [31] and shared culture, norms and goals [32]. There is, however, still a lack of knowledge about how these aspects play out in practice [27].

To gain deeper insight into the normative aspects of integrated, people-centred health services, the concept of values is frequently used. We define values as

“meaningful beliefs, principles or standards of behaviour, referring to desirable goals that motivate action” [33, p.2]

or in laymen’s terms ‘what is important to us’ [34,35]. Often cited conceptual frameworks and taxonomies of integrated, people-centred health services, such as the National Health Service (NHS) typology of healthcare integration [36] and the RMIC [15], describe the role of shared values as crucial [36]. Other integrated, people-centred health services studies relate shared values among actors to the performance of teams and organisational culture [37], professional decision-making [38] and staff commitment [39].

The World Health Organisation (WHO) stresses the need for a unifying values framework in its ‘Global strategy on people-centred and integrated health services’ [1] and the ‘European Framework for Action on Integrated Health Services Delivery’ [40], and proposes core principles to guide the development of people-centred and integrated health services. These principles are based on expert feedback [1,41]. To develop a more scientific basis for a values framework, we refined the list of values by performing a systematic review of the literature [33] and an international Delphi consensus study [42], which resulted in a core set of 18 values (see Table 2). Our Delphi study demonstrated that an international consensus could be reached about a set of 18 values underpinning integrated, people-centred health services as a concept. However, it does not establish whether different actors in different contexts across Europe place different priorities on the individual values. Understanding similarities and differences could be useful for policy makers pursuing policies in support of integration of health services.

**Table 1:** Characteristics of the respondents.


	TOTAL	SERVICE USERS AND INFORMAL CARERS	PROFESSIONALS	POLICY AND DECISION MAKERS	RESEARCHERS

**N**	1,013	163	295	279	276

**(%)**	100.0	16.1	29.1	27.5	27.2

**European sub-region of origin (%)**					

Western Europe	24.6	30.1	15.6	27.2	28.3

Northern Europe	37.7	39.9	33.2	40.5	38.4

Southern Europe	30.1	20.9	47.1	22.9	24.6

Eastern Europe	7.6	9.2	4.1	9.3	8.7

**Years of experience (%)**					

0–5 years	11.5	7.8	8.1	11.6	17.2

5–10 years	13.2	13.1	12.2	11.2	16.4

10–15 years	14.4	13.7	10.8	14.8	18.2

15–20 years	14.5	11.1	13.6	17.3	14.6

20+ years	46.3	54.2	55.3	45.1	33.6

**Gender (%)**					

Male	37.2	30.2	39.1	45.8	30.5

Female	62.6	69.8	60.5	53.8	69.5

Non-binary	0.2	0.0	0.3	0.4	0.0

**Age (SD)**	48.4 (12.0)	53.8 (14.1)	47.9 (11.3)	48.8 (11.0)	45.2 (11.3)


**Table 2:** Priority values and importance scores (n = 1,013).


	INCLUDED IN SELECTION OF 3 MOST IMPORTANT VALUES (%)	IMPORTANCE SCORE ON 1–9 SCALE

**Person-centred** – Valuing people through establishing and maintaining personal contact and relationships, to ensure that services and communication are based on the unique situations of users and informal carers.	47.3	8.15

**Co-ordinated** – Connection and alignment between users, informal carers, professionals and organisations in the care chain, to reach a common focus matching the needs of the unique person.	34.1	8.55

**Holistic** – Putting users and informal carers in the centre of a service that is ‘whole person’ focused in terms of their physical, social, socio-economical, biomedical, psychological, spiritual and emotional needs.	24.7	8.14

**Effective** – Ensuring that care is designed in such a way that outcomes serve health outcomes, costs, user experience and professional experience.	22.8	7.76

**Trustful** – Enabling mutual trusting between users, informal carers, communities, professionals and organisations, in and across teams.	21.3	8.35

**Empowering** – Supporting people’s ability and responsibility to build on their strengths, make their own decisions and manage their own health, depending on their needs and capacities.	18.0	8.28

**Respectful** – Treating people with respect and dignity, being aware of their experiences, feelings, perceptions, culture and social circumstances.	17.8	8.17

**Led by whole-systems thinking** – Taking interrelatedness and interconnectedness into account, realising changes in one part of the system can affect other parts.	15.2	8.10

**Efficient** – Using resources as wisely as possible and avoiding duplication.	15.2	7.98

**Preventative** – There is an emphasis on promoting health and wellbeing and avoiding crises with timely detection and action by and with users, informal carers and communities.	14.7	8.00

**Shared responsibility and accountability** – The acknowledgment that multiple actors are responsible and accountable for the quality and outcomes of care, based on collective ownership of actions, goals and objectives, between users, informal carers, professionals and providers.	13.5	8.29

**Continuous** – Services that are consistent, coherent and connected, that address user’s needs across their life course.	13.4	8.38

**Collaborative** – Establishing and maintaining good (working) relationships between users, informal carers, professionals and organisations – by working together across sectors, and in networks, teams and communities.	12.3	8.24

**Co-produced** – Engaging users, informal carers and communities in the design, implementation and improvement of services, through partnerships, in collaboration with professionals and providers.	9.2	8.34

**Comprehensive** – Users and informal carers are provided with a full range of care services and resources designed to meet their evolving needs and preferences.	8.0	7.96

**Flexible** – Care that is able to change quickly and effectively, to respond to the unique, evolving needs of users and informal carers, both in professional teams and organisations.	7.5	8.22

**Transparently shared** – Transparently sharing of information, decisions, consequences and results, between users, informal carers, professionals, providers, commissioners, funders, policy-makers and the public.	4.2	7.62

**Reciprocal** – Care is based on interdependent relationships between users, informal carers, professionals and providers, and facilitates cooperative, mutual exchange of knowledge, information and other resources.	0.6	7.93


In this explorative study we address this knowledge gap by examining the priority values of different actor groups across different European sub-regions, using the set of 18 values underpinning the concept of integrated, people-centred health services developed in our previous studies [33,42]. This study assesses the importance of these values as perceived by the key actors involved in integrated, people-centred health services, including service users, informal carers, professionals, policy- and decision makers and researchers. These actors have diverse backgrounds, roles and perspectives [43,44,45,46]. The theory of values states that such (sub)groups of people often vary in the relative importance they attach to particular values, also known as value hierarchies, which may guide their behaviour [34,35,47]. To gain insight into their behaviours and preferences, this study examines which values are considered most important by which actors. The study also explores possible variations among European sub-regions. Since integrated, people-centred health services are being implemented in numerous countries and settings [48,49,50,51,52], within a variety of social, economic, political, legal and health system contexts [26,53], we assess whether there are differences in the values that are prioritised by respondents in different European sub-regions. Lastly, we explore possible relationships among the values prioritised by the respondents.

In sum, this study aims to advance the conceptual understanding of the values underpinning integrated, people-centred health services by examining four research questions: (a) How do European actors involved in integrated, people-centred health services assess the importance of the 18 values of integrated, people-centred health services? (b) What, if any, differences may be observed among the values prioritised by different actor groups? (c) What, if any, are the differences among the values prioritised across different European sub-regions? (d) Do relationships exist among the values prioritised by all respondents?

## Methods

### Participants and data collection

The questionnaire was distributed among European service users/patients and informal carers (and their representatives), healthcare professionals, policy and decision makers, and researchers in integrated, people-centred health services with a good understanding of the English language. The actors were invited via the panels of official platforms and associations, both European and national, such as the International Foundation of Integrated Care (IFIC), the European Health Management Association (EHMA), European Patients Forum (EPF) and the AGE Platform. Most of these panels are representative for country, background, gender and other characteristics. The online questionnaire was fielded between October 2019 and January 2020. A total of 1,013 respondents from 42 European countries were included.

### Measures

Participants were asked to fill out the questionnaire from the perspective of their actor role in integrated, people-centred health services. Respondents with multiple roles were asked to choose the role that most closely fits their current situation or position. First, respondents answered questions about their background including: (a) residential country, (b) actor role, (c) years of experience in health services, (d) gender, and (e) age. Second, after a brief explanation about the concept of values, the set of 18 values developed through a systematic review of the literature [33] and an international Delphi study [42] was used to measure priority values. Respondents were requested to indicate the importance of each of the 18 values from their perspective, by awarding a score on a nine-point scale (1 = completely unimportant, 9 = highly important). Then, the participants were asked to select the three most important values out of the set of 18.

### Analysis

The data were analysed using SPSS Statistics for Windows, version 26.0 (International Business Machines (IBM) Corporation, Armonk, NY, United States). For assessment of the nine-point scale, the mean scores and interquartile ranges (IQRs) for each value were calculated to construct scores that reflect their importance. For the three most important selections, the percentages per value were calculated. Binary logistic regressions, including a multi-level model check, were conducted to investigate differences among actor groups and European sub-regions. Respondents were clustered in four actor groups: (a) service users/patients and informal carers, (b) professionals, (c) policy and decisionmakers, and (d) researchers.

Countries were clustered in four European sub-regions: (a) Western Europe, (b) Northern Europe, (c) Southern Europe, and (d) Eastern Europe. This grouping is based on the United Nations Geoscheme for Europe [54]. Multi-collinearity among independent variables was examined using Cramer’s V, chi-square and Spearman’s rho tests. For actor groups we controlled for sub-regional origin, gender, age and years of experience. European sub-regional control variables included actor group and gender. To investigate relationships among the 18 values, a principal components factor analysis (PCA) was conducted. The rotation method was Oblimin with Kaiser Normalization. The number of factors was based on the number of Eigenvalues ≥ 1.0 in the PCA. The two factors were interpreted by the six researchers based on the ranking of values per factor, considering conceptual patterns. Disagreements were resolved by discussion.

### Ethical considerations

The study design as described above has been reviewed and approved by the Ethics Review Board of Tilburg School of Social and Behavioural Sciences (Ref. ‘EC-2019.EX153’). Participation in this research was entirely voluntary. All respondents gave informed consent prior to participation in the study and were free to decline to answer a particular question for any reason. Survey answers were collected and stored through secured and password protected electronic software. No identifying information such as IP addresses or names was collected. All respondents remained anonymous.

## Results

### Demographics

Table 1 shows the characteristics of the respondents. In total, 1,013 respondents from four actor groups and four European sub-regions completed the survey. Most respondents had twenty years of experience or more (46.3%) and were female (62.6%). The respondents had an average age of 48.4 years (SD = 12.0).

### General importance scores

The mean importance scores of the values ranged from 7.62 to 8.55 on a scale from 1 to 9. ‘transparently shared’ was rated the lowest (mean 7.62, IQR 7-9) and ‘co-ordinated’ (mean 8.55, IQR 8-9) received the highest importance score. The percentages of the values selected as one of the three most important values out of the 18-item set ranged from 47.3% to 0.6%. ‘Reciprocal’ was the least selected value (0.6%) and ‘person-centred’ was included by most of the respondents (47.3%).

### Actor groups

**Figure 1:**
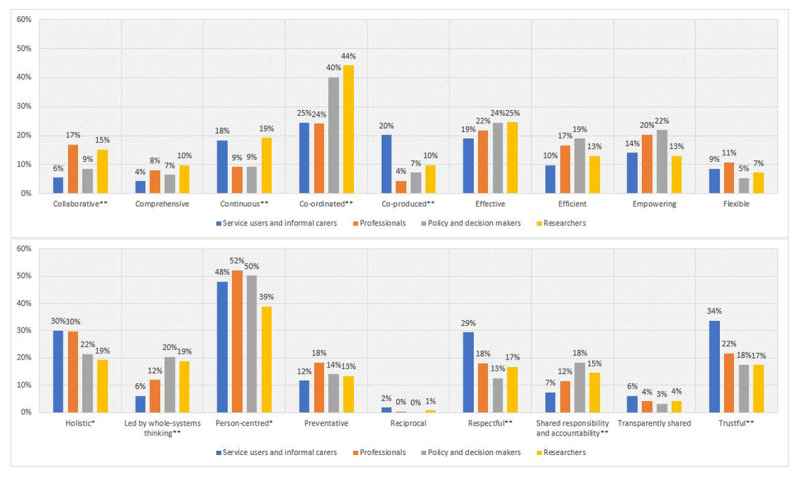
Priority values of the actor groups. * p ≤ 0.05, ** p ≤ 0.01.

After controlling for sub-regional origin, gender, age and years of experience, binary logistic regression identified statistically significant effects of the actor group of respondents for the prioritisation of ten values. Figure 1 summarises the association of the actor group with the priority values. Statistically significant effects are indicated by an asterisk (* p ≤ 0.05, ** p ≤ 0.01).

Controlled binary logistic regression demonstrated that service users and informal carers were significantly more likely to select the values ‘co-produced’, ‘respectful’ and ‘trustful’ as one of their three most important values, than all the other actor groups (all p-values ≤.033). Service users and informal carers, and researchers were significantly more likely to select ‘continuous’ than professionals and policy and decision makers (all p-values ≤.034).

Professionals, service users and informal carers were significantly more likely to include ‘holistic’ in their selection of three most important values than the other actor groups: policy and decision makers and researchers (all p-values ≤.020). Furthermore, professionals and researchers were more likely than the other actor groups to select ‘collaborative’ as one of their three most important values (all p-values ≤.002).

Policy and decision makers and researchers were more likely than the other actor groups to include ‘co-ordinated’ and ‘led by whole systems thinking’ in their selection of the three most important values (all p-values ≤.001). Furthermore, policy and decision makers and researchers were more likely to select ‘shared responsibility and accountability’ as one of their three most important values than service users and informal carers (all p-values ≤.015).

### European sub-regions

**Figure 2:**
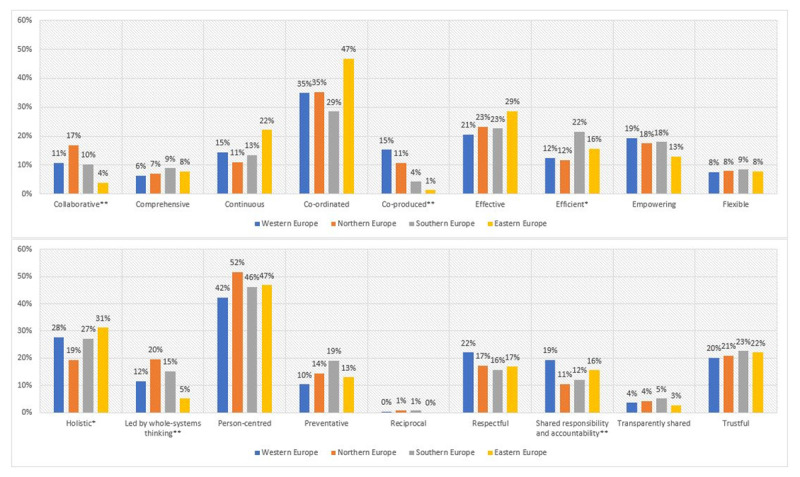
Priority values across European sub-regions. * p ≤ 0.05, ** p ≤ 0.01.

After controlling for actor group and gender, binary logistic regression demonstrated statistically significant effects of the sub-regional origin of respondents for six values associated to integrated, people-centred health services. Figure 2 displays the association of sub-regional origin with the priority values. Statistically significant effects were found for ‘collaborative’, ‘co-produced’, ‘efficient’, ‘led by whole systems thinking’, ‘holistic’ and ‘shared responsibility and accountability’ (all p-values ≤.023), which are indicated by an asterisk (* p ≤ 0.05, ** p ≤ 0.01).

In comparison to other respondents, Western European respondents ranked ‘efficient’ (12%) relatively low, but ‘co-produced’ (15%) and ‘shared responsibility and accountability’ (19%) relatively high. Northern European participants awarded lower scores to ‘shared responsibility and accountability’ (11%), ‘efficient’ (12%) and ‘holistic’ (19%), while assessing ‘collaborative’ (19%) and ‘led by whole systems thinking’ (20%) relatively higher. Respondents from Southern Europe rated ‘efficient’ (22%) especially high. Eastern European respondents attached relatively lower scores to ‘co-produced’ (1%), ‘collaborative’ (4%) and ‘led by whole systems thinking’ (5%), and relatively higher scores to ‘holistic’ (31%).

### Relationships between values

The PCA resulted in a two-factor solution with a cumulative loading of 52.8%. The analysis revealed two clusters of values. Ten of the 18 values loaded on the first factor. The remaining eight values all loaded on the second factor (see Table 3). Factor 1, labelled as ‘people related’, mainly consists of items related to how people interact acknowledging a worthwhile relationship between a person and a health professional. Factor 2, labelled as ‘governance and organisation’ contains items referring to the governance and organisation of integrated, people-centred health services and to what it should contribute.

**Table 3:** Results of PCA and Oblimin with Kaiser Normalization of the two factors.


	--- FACTOR ---	

	PEOPLE RELATED	GOVERNANCE AND ORGANISATION

	**Eigenvalues and cumulative proportion of variance explained by principal components analysis**	

Eigenvalue	8.402	1.026

Cum. variance	46.678	52.375

	**Factor pattern Oblimin with Kaiser Normalization**	

Empowering	**.805**	.064

Holistic	**.790**	.064

Person-centered	**.781**	.048

Co-produced	**.701**	–,.70

Respectful	**.608**	.015

Trustful	**.536**	–.124

Collaborative	**.528**	–.262

Shared responsibility and accountability	**.438**	–.241

Reciprocal	**.423**	–.415

Flexible	**.408**	–.359

Effective	–.179	**–.908**

Efficient	–.105	**–.877**

Continuous	.142	**–.697**

Transparently shared	.190	**–.556**

Preventative	.236	**–.552**

Led by whole-systems thinking	.237	**–.493**

Comprehensive	.331	**–.472**

Co-ordinated	.288	**–.465**


## Discussion

In this explorative study, we measured the value importance scores and selections of the three most important values of 1,013 actors across Europe to identify their priority values. We found that each of the 18 values associated to integrated, people-centred health services is considered important, receiving high ratings from every actor group from all the European sub-regions. With scores ranging from 7.62 to 8.55 on a nine-point scale, the absolute differences among these scores are small, indicating an absence of an overarching value hierarchy among all values. Each of the values in the set is considered important from all the included actor perspectives and geographical contexts, which confirms the consensus reached in our previous Delphi study [42].

These similarities notwithstanding, the study did find statistically significant differences in the relative importance of different value scores across different actor groups, suggesting a distinction in emphasis that could be expected considering the different roles, responsibilities and perspectives of various actors [43,44,45,46]. Service users and informal carers, for example, attached a significantly higher priority to values, such as ‘respectful’, ‘trustful’ and ‘co-produced’, that express how they ideally would like to experience integrated, people-centred health services and the relationships with their carers. These findings are similar to those found in recent studies on the service user perspective in integrated, people-centred health services.

Recent studies by Kuluski et al [55], Lawless et al [56] and Youssef et al [8] identify several attributes and themes that are important to service users. A major part of these components includes relational aspects [56], such as feeling heard [55,56], feeling respected [56] having someone to count on [55], caring about the person [8] and collaboration with the service user [8]. The priority values of the professionals in our study such as ‘holistic’ and ‘collaborative’, relate to their day-to-day work. Similar values underpin the foundations of interdisciplinary collaboration in the literature [57,58,59].

The policy and decision makers in the study prioritised values referring to how the organisational and governance aspects of integrated, people-centred health services should be shaped. Examples are ‘co-ordinated’, ‘shared responsibility and accountability’ and ‘led by whole systems thinking’. These values can also be found in articles on organising and governing integrated, people-centred health services, which point to collaborative networks as organisational models and focus on relationships across instead of within organisations [60,61,62,63].

The existing literature also highlights the variety of actor perspectives in integrated, people-centred health services. Referring to the work of Nolte and McKee, Goodwin et al. state that the variety of interpretations and definitions of integrated, people-centred health services as a concept may (at least in part) be the result of the different viewpoints of its various actors [45,64]. Similarly, Shaw, Rosen and Rumbold present multiple actor perspectives on integrated, people-centred health services, such as the perspectives of policy and decision makers (policies, regulations, financing arrangements, care systems), professionals (provision and co-ordination of care), and service users and informal carers (experience of access and navigation across the care system, information-sharing) [46]. Goodwin et al. stress that all these interpretations and definitions are potentially legitimate and that integrated, people-centred health services should not be defined narrowly [65]. In line with this statement, our study demonstrates that although actors all seem to support integrated, people-centred health services as a concept, they observe integrated, people-centred health services from different angles and find different components of it particularly important.

These distinctions in emphasis are also demonstrated by the factor analysis in this study, which uncovers two clusters (factors) of values with different accents. The ‘people related’ values accentuate how people want to be treated within the relationship between service users and professionals. The ‘governance and organisation’ values highlight what is considered important in organising and governing health services integration, and to what such integration should contribute. These two clusters could be seen as two sides of the same coin of care integration. Both provide an important additional perspective for the realisation of integrated health services delivery. On the one hand, to pursue ‘people related’ values in integrated, people-centred health services, it is also necessary to work on ‘governance and organisation’ values. Values related to governance and organisation may facilitate an enabling environment supporting the delivery of health services that are people-centred, illustrated by ‘people related’ values such as respectful, trustful and empowering. On the other hand, if we would only base the delivery care services on ‘governance and organisation’ values, this could potentially limit important people related features of integrated health services. It is important to balance between these two clusters, and to accentuate particular values when needed. The existence of these two factors therefore helps us to better understand distinctions in emphasis and how these values together form a whole. In this way, our study results contribute to the often incoherent definition(s) of integrated, people-centred health services, by providing the components for a conceptually based definition.

We see the pursuit of all values within both clusters as essential for realising integrated, people-centred health services. This is in line with existing frameworks [1] and commonly used definitions [2,40,66] stressing that health services integration requires concerted efforts on different levels and among a diversity of actors, around individual persons, families and communities (resembling the people related factor), but also within the broader organisation and governance of services and within regional or national health systems (factor governance and organisation). The insights of our study provide a normative basis for such multi-level pursuits of health services integration and its definition.

For implementation of integrated, people-centred health services, the distinctions in emphasis by the actor groups suggest that we need to recognise and take into account a possible range of multiple values. We elaborate on two aspects of integration that could benefit from taking into consideration the findings of this study: (a) coordination by network facilitators and (b) collaborative attitudes and competencies of actors. First, care integration often takes place in networks of organisations and individuals. A network facilitator or broker, for example a lead organisation or a network administrative organisation (NAO) [62,67], can play an important key connecting role in complex integrated, people-centred health services networks by, for example, resolving conflicts and building trust among actors in the collaborative network [62,68]. Our study adds to this concept by demonstrating that network facilitators would benefit from uncovering and taking into account the values prioritised by all the different actors. Network facilitators should be able to acknowledge and understand what matters to the different actors in pursuing integrated, people-centred health services and how they define particular issues. They need to take the social constructions and perspectives of the collaborating actors into account. Examples of such facilitators are cultural brokers [69] and boundary spanners [70].

Second, our findings suggest that collaborating actors in care integration should also be aware of each other’s values. Many studies demonstrate that interdisciplinary collaboration among professionals requires a collaborative attitude [71,72,73,74,75], described as

“being able and willing to work together with respect for partners” by Janssen et al. [71, p.7].

Our study adds to this concept by showing that such an attitude should also include acknowledging and respecting each other’s priority values. In the Model of Collaboration of D’Amour et al., this awareness of one another’s value orientations is called internalisation, helping actors to see the bigger picture which translates into mutual trust and understanding [76,77]. Our study suggests that normative considerations, such as values, deserve attention in the development and education of collaborative attitudes and competencies [58,78,79].

Compared to the actor group analysis, there are fewer differences among prioritised values across European sub-regions. Significant differences were identified for six values. ‘efficient’, for example, is a highly ranked value in Eastern Europe, while ‘co-produced’ is more highly ranked in Western Europe. This suggests that the context in which integrated, people-centred health services take place matters, as also stated in the work of Busetto [26,80]. Some of the observed differences in sub-regional variations in value scores are likely to reflect differences in the historical context of the countries in these sub-regions. For example, collaboration and co-creation were not values that characterised health services in the former communist countries in the eastern parts of the European region where centrally dictated norms and protocols were the custom, leaving limited room for collaboration and co-creation [81]. Furthermore, reform of the health systems in former communist countries with their heavy reliance on hospital-based care has to a large extent been driven by a focus on improving the efficiency [81]. It is therefore not surprising that respondents in these countries place relatively greater importance on this value than the respondents in countries that have experienced different pressures for reform.

### Practical implications

Our study demonstrates that all respondents consider the values associated to integrated, people-centred health services important. Additionally, it reveals distinctions in emphasis among the values prioritised by actor groups and across sub-regions. These distinctions in emphasis may also exist in practice. Each actor has their own perspectives and interests. The distinctions in emphasis may complement each other, which could lead to a whole that is greater than the sum of its parts. At the same time, actors may have different interpretations of values. It would therefore be helpful to make the priority values of the actors more explicit and tangible. Our set of 18 values can be used as a vocabulary in dialogues and exercises to facilitate different actors to put themselves in the shoes of others, enabling them to examine the values that they find important in collaboration from both a personal and a collective perspective. Once each actor has selected ‘their’ values from the list of 18, the results can be discussed collectively and together the actors can explore what particular values mean to them and to the other actors. Possible differences in the interpretations of the values can also be clarified. For example, what does effectiveness or trust mean to the different partners, how do they apply these values in their daily work, and what do they expect from the other actors? On what values do they agree and disagree, and what does this mean for the collective service? For example, if all actors find efficiency important, what does this imply for service delivery? Box 1 contains a practice example of such a values dialogue. In this way, our set of values could form a shared vocabulary on which actors in integrated, people-centred health services could base their joint efforts.

**Box 1 Practice example of values dialogue**

The health and social care professionals in a Dutch dementia care network engaged in dialogue about their priority values. The majority of them attached most importance to person-centeredness. Their expectation was also that most of them would agree that this is a leading value. However, when the professionals explored the meaning of person-centredness as a value, different interpretations arose. While some professionals associated person-centredness with empowerment and self-management, others explained the concept as acting according to the service user’s preferences. They also noted that service users and their families could have different views on this. In practice, these differences sometimes complement each other, but they could also complicate collaboration. It was therefore helpful to engage in dialogue about values and their meaning. The values were made concrete by using the set of 18 values as a vocabulary, enabling a deeper discussion of the interpretations of different professionals. In this way, professionals were able to step in the shoes others and understand the background of decisions and behaviours of others.

### Strengths and limitations

Several strengths and limitations of this study should be considered when interpreting its findings. A strength of our study is its large number of respondents (n = 1,013). Second, the questionnaire used to assess priority values is based on a systematically developed set of values of integrated, people-centred health services [33,42]. A first study limitation is the varying number of respondents among both actor groups and European sub-regions. Second, the online questionnaire was administrated in English. Although English is a widely spoken language in Europe, it is not the native language of all respondents, which could have led to differences in interpretation of the questions. Furthermore, the results may not be representative of non-English speakers in the regions.

### Future research

Multiple recommendations for future research can be made. First, although our study revealed relevant insights into priority values, it did not consider how these value orientations are constructed and what factors determine this. It would be valuable to better understand the factors behind the differences we found in this study, which could be achieved by in-depth qualitative research including methods such as focus groups and interviews. Second, as we recommend using our set of values as a vocabulary to engage in dialogue, it would be interesting to empirically study how this exchange might take place. By performing case study research, we could observe how actors exchange values and how this influences integration. These insights may reveal concrete opportunities for improving integrated, people-centred health services by using values as a vocabulary. Third, it would be worthwhile to gain more insight into how a values-driven perspective can drive the implementation of integrated, people-centred health services. We should investigate how a values-driven approach – as a methodology, e.g., alliance building and network development – can inform implementation and improvement in practice. This could be done by conducting empirical case studies.

## Conclusion

Although much knowledge about the functional aspects of integrated, people-centred health services is available, the actual implementation of integrated, people-centred health services remains challenging. More insight into the normative dimension of integrated, people-centred health services is needed. The attention paid to values in integrated, people-centred health services has therefore increased. However, there is still a lack of information on how these values are prioritised by different actor groups across different European sub-regions. Our study confirms that the set of 18 values underpinning integrated, people-centred health services is considered important by all the participants in the study.

Additionally, our study documents that there are distinctions in emphasis among the values prioritised by actor groups and across sub-regions. Furthermore, our study reveals two clusters of values: ‘people related’ values and ‘governance and organisation’ values. Our study suggests that when integrating healthcare services, for example within co-ordination and collaboration processes, normative considerations, such as priority values, deserve more attention. A dialogue is needed to make the values of the different actors more explicit, to acknowledge the priority values of others, and to use them as a basis for promoting integrated, people-centred health services more effectively. Our set of 18 values underpinning integrated, people-centred health services can be used as a vocabulary for such a dialogue and the conceptual definition of integrated, people-centred health services.

## Data Accessibility Statement

Individual-level survey data generated during and/or analysed during the study are not publicly available due to ethical and confidentiality reasons (Ref. ‘EC-2019.EX153’). Subregional, country and actor group level data are available from the corresponding author on reasonable request.

## Abbreviations

EHMA: European Health Management Association

EPF: European Patients Forum

IBM: International Business Machines

IFIC: International Foundation of Integrated Care

IQR: Inter Quartile Range

NAO: Network Administrative Organisation

NHS: National Health Service

PCA: Principal Components Analysis

RMIC: Rainbow Model of Integrated Care

SD: Standard Deviation SD

SCGS: Catalan Society of Healthcare Management

SEDISA: Sociedad Española de Directivos de la Salud

WHO: World Health Organisation
